# Cervical cancer screening and vaccination acceptability and attitudes among Arab American women in Southeastern Michigan: a qualitative study

**DOI:** 10.1038/s41598-024-64462-1

**Published:** 2024-06-13

**Authors:** Lilah Khoja, Heidi G. Torres, Layla Hak, Manar Aljebori, Minh Tung Phung, Andrea Sakleh, Matthew Stiffler, Madiha Tariq, Celeste Leigh Pearce

**Affiliations:** 1 Department of Epidemiology, School of Public Health, University of Michigan, 1415 Washington Heights, Ann Arbor, MI 48109 USA; 2 Center for Arab Narratives at the Arab American National Museum, ACCESS, 6450 Maple St., Dearborn, MI 48126 USA; 3 ACCESS Community Health and Research Center, 6450 Maple St., Dearborn, MI 48126 USA

**Keywords:** Cervical cancer screening, Arab American, HPV, Vaccination, Cervical cancer, Population screening, Epidemiology, Preventive medicine

## Abstract

Among Arab-American women in Michigan, rates of cervical cancer screening are lower than those in non-Hispanic White and Black women in the state. A deep understanding of the Arab community’s perspective on cervical cancer screening is needed to address the disparity in rates across populations in Michigan. Arab and Chaldean women across Michigan were invited to participate in Zoom-based focus groups to understand the attitudes, acceptability, and barriers of cervical cancer screening among this population. Four focus groups with a total of 19 women aged 30 to 61 were conducted. The focus groups were conducted in English, Arabic, or both languages. The guided discussion was focused on knowledge of cervical cancer and Human papillomavirus (HPV) and its transmission, attitudes towards HPV vaccination, and attitudes towards cervical cancer screening. HPV self-sampling as an alternative to traditional provider-based screening was specifically discussed as this has been proposed as a way to increase screening in hard-to-reach populations. The conversations revealed insights related to barriers at the individual and community levels for screening and vaccination, attitudes towards preventive health care including screening, a need for accessible women’s health literature, and health education. The women also discussed vaccine hesitancy related to HPV and COVID-19, suggesting a need for targeted community interventions.

## Introduction

Cervical cancer is the most diagnosed gynecologic cancer amongst women worldwide, with an estimated 604,000 new cases and 342,000 deaths in 2020^1^. Cervical cancer is preventable through a combination of primary (vaccination) and secondary (screening) prevention followed by treatment of precancerous lesions^2–4^. In the United States, the Papanicolaou (Pap) test alone or in conjunction with *Human papillomavirus* (HPV) testing for cervical cancer screening has been successful in reducing incidence and mortality by ensuring appropriately timed treatment^5^.

Despite these reductions, there are substantial racial/ethnic disparities in Pap screening uptake in the United States^6,7^, with non-Hispanic White women being more likely to receive Pap tests and co-testing with HPV than Black women or Hispanic women^6,8^. These studies tend to obfuscate women who identify as Arab due to the classification of Arab Americans as non-Hispanic Whites. In Michigan, however, the Behavioral Risk Factor Surveillance System (BRFSS) specifically conducted two surveys among the Arab population in the state^9,10^.

Southeastern Michigan has the highest concentration of Arab and Arab Americans (hereafter referred to as Arab American) in the United States; Michigan's Arab American community includes a large number of Chaldeans, a Christian community mainly from Iraq. Cervical cancer screening rates among Arab American women in Michigan are consistently lower than that of non-Hispanic White and Black women in the state^9,10^. In 2016, the Michigan BRFSS Arab American-specific survey found that among screening-aged Arab American women, only 62.2% reported ever having a Pap test compared to 90.5% of non-Hispanic White, 83% Black women, and 78% Hispanic women. Moreover, only 54.9% of Arab American women in Michigan had appropriately timed Pap screening whereas the proportions were 73.5%, 74.8%, and 63.3% for non-Hispanic White, Black, and Hispanic women, respectively. The reasons for these lower screening rates among Arab American women are unclear. Further, a quantitative study carried out in Dearborn, Michigan in 2011 among women receiving services from the Arab Community Center for Economic and Social Service (ACCESS) found that unmarried women, those without a doctor’s recommendation, and those with competing priorities were less likely to be screened for cervical cancer^11^. A second quantitative study also carried out in Dearborn, Michigan in 2019 found that those who lack insurance and those who had been in the United States for less than 10 years were less likely to be screened for cervical cancer^12^.

Addressing the low cervical cancer screening rates among Arab American women in Michigan requires a deep understanding of the community’s perspective on cervical cancer prevention and screening. Thus, we conducted a qualitative study involving a series of focus groups in Michigan to understand the attitudes, acceptability, and barriers to cervical cancer screening among, as well as attitudes towards HPV vaccination Arab American women. This work provides insights into current views and approaches that may be successful in increasing cervical cancer screening uptake in this population.

## Methods

The focus groups were conducted as a part of the Study of Arab American Health Attitudes (SAHA), which aims to study Arab American health disparities. These focus groups were planned as a qualitative arm of a currently underway study (SAHA-HPV) which is meant to evaluate the feasibility and acceptability of the use of self-sampling devices for cervical cancer screening instead of traditional screening methods, such as the Pap test.

This study was approved by the University of Michigan Health Sciences and Behavioral Sciences Institutional Review Board (IRB-HSBS) and granted a determination of exemption under Federal Exemption 2, which does not require signed informed consent (HUM00150710). All research was performed in accordance with relevant guidelines. All potential focus group participants were provided with a study informational sheet outlining the study goals, benefits, and risks in Arabic and English one week ahead of the scheduled focus group time. Upon the start of each focus group, the study team discussed the information sheet with each participant privately and ensured that each participant gave informed verbal consent prior to participating in the focus groups. Participants received a $75 gift card for participating. Identifiable information was only collected to ensure participants received their incentives and was destroyed once the gift card was mailed.

### Study design

The purpose of this study was to gather information on the attitudes and beliefs that Arab American women who reside in Michigan have about cervical cancer risk, screening, and vaccination against HPV. Any woman who identified as Arab, Arab American, and/or Chaldean aged between 30 and 65 (i.e., those eligible for cervical cancer screening via HPV testing^13^) was invited to participate in the focus groups. Participants were recruited through women’s only beauty salons in Dearborn, MI; via ACCESS social media channels and newsletters; and through community social media pages on Instagram. The goal was to recruit 3–6 women per focus group. The focus group sizes were kept small based on best practices for Zoom-held studies, due to the potentially sensitive nature of the topics, and the tight-knit nature of the community^14–16^.

Four focus groups were conducted via Zoom^17^ during the summer of 2021. One focus group was conducted solely in Arabic, two in English, and one in a combination of both languages. Women completed a brief anchoring survey with demographic questions and a self-assessment of HPV knowledge. The focus group guide provided an overall structure for the focus groups and covered beliefs about cervical cancer and HPV risk, attitudes towards traditional methods of cervical cancer screening (i.e., Pap smears), self-collection sampling methods for cervical cancer screening, screening uptake, attitudes towards HPV vaccination, and barriers that may exist for screening and vaccination. The Health Belief Model and Social Cognitive Theory were used in developing the focus group guide^18^.

### Data processing and analysis

The number of women in each focus group was five, four, seven, and three. All participants identified as Arab, Arab American, and/or Chaldean. Focus group recordings were transcribed, translated if necessary, and uploaded to the qualitative software platform, Dedoose Version 9.0.17^19^. Three members of the study team engaged in the coding and analysis of the focus groups (LK, HG, and LH). An inductive approach to coding was employed. Initially, one focus group was coded using a collaborative open coding process with descriptive codes^20^. A discussion was carried out to determine suitable codes and definitions, and the agreed-upon codes were then used to create a codebook. This focus group was then recoded, and the codebook was further revised after the emergence of new codes. The remaining three focus groups were then coded using this codebook. Code reports were prepared. Ultimately, the codebook included 38 primary codes with 12 sub-codes; a total of 1,216 codes were applied to 886 excerpts. Thematic content and ethnographic analysis techniques were applied to identify patterns and themes across several meetings^21^. Inter-coder reliability was assessed qualitatively throughout the process^22^.

## Results

The four focus groups consisted of 19 women from across Michigan (see Table 1). The mean age of participants was 37.9, and 61.1% of the participants were married. The anchoring survey was completed in English by 68.4% of the participants, however, only two of the focus groups were conducted entirely in English. More than half of the participants indicated that they were “not very well-informed” about cervical cancer (55.6%); only 11.1% indicated that they were either well-informed or very well-informed.

**Table 1:** General characteristics of participants (N* = 19).

	N (%)	
Mean Age (N = 17)	37.9	8.7 (SD)
Married	11	61.1%
Survey Language		
Arabic	6	31.6%
English	13	68.4%
How well would you say the financial needs are met in your household?		
More than sufficiently met	3	16.7%
Just sufficient	9	50.0%
Barely making it	3	16.7%
Not met at all	3	16.7%
How well-informed do you think you are about cervical cancer?		
Not informed at all	1	5.6%
Not well-informed	10	55.6%
Moderately informed	5	27.8%
Well-informed	1	5.6%
Very well-informed	1	5.6%
Had vaginal sex (intercourse) before	15	83.3%
Had oral sex before	8	47.1%
Has children	15	83.3%

*Complete data available for 17 participants; partially complete data available for 1 participant; and missing data for 1 participant.

### Risk factors

Initial discussions in the focus groups centered on their knowledge of risk factors for cervical cancer as well as attitudes related to screening for the disease. Participants described a range of risk factors that they believed could lead to cervical cancer, including menopause and age, hormonal changes due to stress from war, genetics, diet, and a weakened immune response. A list of the topics and themes explored are presented in Table 2. Representative quotes surrounding these beliefs included the following:

**Table 2:** Topics and themes discussed and explored.

Cervical cancer risk factors
Menopause and age
War and hormonal changes
Genetics
Diet and immunity
HPV and sexual behaviors
Neglect
Attitudes towards cervical cancer screening
Discomfort
Embarrassment
Concern about results
Responsibility
Regular Testing
Unaware
Fear
Lack of health literacy as a barrier
Awareness
Understanding
Ambivalence towards vaccination
Vaccine hesitancy
Lack of vaccine awareness
Stigma
Areas of potential intervention for screening and vaccination
Favor self-collection
Concerned about self-collection
Increasing Awareness
Fatalism

Menopause: *“I believe women who are aged 50 or older, the ones who have gone through the natural change who can't bear children anymore, these are the people I believe to be most at risk for cervical cancer.” (Group 3).*

Hormones: *“I believe oftentimes there are issues with the hormones. This is what I believe, you know this is because of maybe wars, or other traumatizing events, or even personal changes which can lead to hormonal changes, but these things can lead to these diseases”. (Group 3).*

Genetics: *“It runs in the family.” (Group 3).*

Immune system: “*A weakened immune system also makes you prone to this, for example if you eat unhealthy food, your immune system will be weakened, which will make you more likely to get sick.”(Group 3).*

Participants also identified HPV, multiple sexual partners, and unsafe sexual practices as risk factors for cervical cancer. They also expressed that those who are sexually active are especially at risk for cervical cancer if they were not get vaccinated.

Sexual activity: *“I do not have thoughts but like I have heard. I am not sure. If through, if you have like more than one partner or you are having sex with different people. That this would increase your rate of having cervical cancer.” (Group 2).*

Vaccination: *“Anybody that didn’t get the vaccine, and especially if they’re sexually active.” (Group 4).*

### Barriers

Most of the participants were familiar with and had done a Pap test in the past. They felt that neglecting your health puts you at risk for cervical cancer. They shared their experiences with and feelings about having a Pap test. Overall, the women had negative associations with Pap tests, citing discomfort, embarrassment, and anxiety over waiting for results. Particularly, the embarrassment stems from the state of undress and position required for a Pap test, especially in front of a male physician. However, for women who were up to date on their screening, discomfort did not stand in the way of their scheduling screening test.

Neglect: *“I believe the people most at risk for this disease are the ones who do not maintain their health, the ones who do not do their yearly exams or failure to do diagnostic tests that have to be done yearly.” (Group 3).*

Discomfort: *“And you know, it just-, also just the-, I feel like I can feel it. The scraping feeling so I have to prepare myself mentally when I do go to a Pap Smear.” (Group 1).*

Embarrassment: *“Okay, so I feel like I have to prepare myself mentally for it, because, you know [laughs], I’m just opening up my legs for a doctor.” (Group 1).*

Anxiety: *“But honestly, I feel a lot of anxiety and nervousness. By the time the results come out, I feel as though my soul has left my body (laughs), but I say thank God.” (Group 3).*

Commitment to screening*: “But I feel like this is responsibility for myself to kind of-, you know, it’s a way of protection.” (Group 1).*

Additionally, participants gave various reasons for not being current with or ever having had cervical cancer screening, including being too busy to keep up regularly and fear. However, there was a pervasive belief that screening was only needed when pregnant.

Busy: *“I do not have any reason. It is kind of I am bit lazy going to the doctor and also, I work and have kids. And all this, but I am kind of- I should be doing more regular check-ups.” (Group 2).*

Fear: *“I am too scared, honestly, to get one done. From what I understand, there are so many benefits to getting screened early, and capturing cancer early because it makes treatment easier, but I feel a lot of fear and anxiety. Basically, a difficult emotional state. So maybe that’s why I’ve never gotten one done.” (Group 3).*

Pregnancy: *“Like I only thought like you needed a Pap smear only if you are pregnant and like I know when I am not pregnant, I am like eventually when I have that check-up. Like oh you are overdue for a Pap smear. But I was like-, I was not educated enough about what exactly the Pap smear does.” (Group 2).*

*“Once I am pregnant, I actually go and then, I get all the tests that need to be done. And then I call it a day.” (Group 2)*

Additionally, three major themes emerged from the analysis: (1) lack of health knowledge and health literacy; (2) ambivalent attitudes towards vaccination; and (3) areas of potential intervention for screening and vaccination.

## Lack of health knowledge and health literacy

In addition to trying to understand what the participants’ understanding of cervical cancer and its risk factors were, they were also asked about cervical cancer screening practices, both theirs and within the community. The focus group moderators shared with the participants the low cervical cancer screening rates in the Arab American community in Michigan^9,10^, and none of the participants were particularly surprised. The participants identified this lack of awareness and a lack of women’s health literacy as a major barrier to screening uptake amongst the women in their communities. Several participants expressed a belief that this is more prominent among immigrants, who may have not had access to Pap tests or a formalized health education:

Immigration and health literacy: *“For me, I am from Iraq, we do not—we do not have an awareness of these diseases. We do not have awareness about girls and women’s health. By the time these diseases are diagnosed, it is so late. When I came to the US that's when I heard about this and started getting tested, I was afraid.”* (Group 3).

*“There are some women who if you tell them they have to do these tests, they say well back in my country I never did those tests and there was nothing wrong with me, so why do it now or tell anyone to do it now?” (Group 3).*

Lack of health literacy: *“There isn't anything about women's health and the health needs of women and their children, there just isn't an awareness that these things are important for women, maintaining health is important to let women live their lives, which could be taking care of her husband and raising her kids. But there is just no awareness, there needs to be more.” (Group 3).*

*“There just isn't awareness, medical centers, awareness programs, doctors, or someone to tell us about our girls’ health, our boys health. Especially our girls. If we do not know, how will we be able to teach them?” (Group 3).*

*“They don't understand – they don't know the reality of these diseases. They do not see a need to go and get tested.” (Group 4)*

### Ambivalent attitudes toward vaccination

Part of the discussion revolved around vaccination against HPV—participants were asked if they had heard of the HPV vaccine, and if they had children, whether their children were vaccinated. If the participants did not have children, they were asked if they would vaccinate their children. The responses were mixed. While some participants were very vocally supportive of the vaccine for all their children, others did not know that they could vaccinate their sons, and still others were not even aware of the vaccine. Some participants noted that there exist pockets of vaccine hesitancy amongst the Arab American community in Michigan. This anti-vaccine sentiment was shared by some participants in the focus groups. Many participants spoke of vaccination, broadly, and discussed the HPV vaccine in relation to the COVID-19 vaccine. They also discussed vaccines that are regularly administered to children and those immigrating to the United States. Some participants also mentioned initial concerns about the vaccine due to the stigma around sexual behavior, which they said is also reflected in community attitudes.

In favor of vaccination: *“When the doctor told me [about the vaccine], I said of course. Anything, you know, we want to protect our kids from everything.” (Group 4).*

*“I did not realize it was meant for boys until he explained, and if anything, that is going to cause–you know–some kind of protection. I was for it, so I gave it to him.” (Group 2).*

*“I wasn't aware—the doctor called me and said she had a shot that she had to take. So I asked them, what shot is this? They told me, and I said ok, no questions asked as they say. This is protection for my daughter.” (Group 4).*

Unaware of vaccination: *“To be honest, I have no idea what vaccinations my sons have gotten. My son is 16, he got some vaccines then. They get all the vaccines, but I will be asking the doctor, my children’s doctor, if they’ve taken the [HPV] vaccine and if they need to take it. If she says yes, then of course, they will get the vaccine. Thank you so much for bringing this to my awareness. Because we did not know.” (Group 3 ).*

*“I never heard of a vaccine–HPV vaccine. Even the doctor never mentioned it.“* *(Group 1)*

Vaccine hesitant: *“I see it like cases where someone take it and then like there is a big reaction to it. And that is why I did not [vaccinate my children].” (Group 2).*

*“If I had a daughter, I don't think I'm gonna push for any vaccination until they make their own choices. So, like my son, I don't-, I'm not gonna vaccinate him, but if he decided later on, he wanted to take it, it's up to him. Again, I-, as the last question I mentioned, I'm not really a fan of vaccinations so that's why I'm not gonna push my son for any vaccinations.” (Group 1).*

Stigma related to sexual behavior: *“Me personally, I was against my daughter getting it just because I was not educated enough on it and I did feel like-, well she is not going to be sexually active at a young age nor should she have to take it. But her pediatrician–you know–had a very thorough discussion with me. And then I ended up having her do it.” (Group 2).*

## Areas of potential intervention for screening and vaccination

The participants, regardless of their stance on vaccination, were all eager to discuss ways to improve the health of their communities. HPV self-collection was discussed as a strategy to improve screening uptake. The participants also spoke to the importance of improving health literacy and increasing awareness. They also discussed the need to combat fatalism.

### Self-collection

Participants were shown pictures of several HPV self-collection devices^23^ and asked to comment on whether they would consider using them in lieu of a Pap test. Attitudes towards self-collection were mixed. Some women were enthusiastic, citing a lack of embarrassment and the flexibility to do the test on their own time.

Lack of embarrassment: *“It seems really nice – using it at home and then popping it in the mail. No embarrassment, without cost. It seems comfortable, you know what I mean? I'm for the device.” (Group 4).*

*“Revealing myself that way to the doctor is embarrassing for me. That's it. That's why I would use the device.” (Group 3)*

Convenience: *“I possibly might, it is more convenient for me because I am at home versus having to go to the doctor. And if-, and if I am concerned about something, I can always talk to my doctor”(Group 2).*

Concerns about properly using device: *“Personally, I would rather just go to the doctor, because its better. It's more safe that way. I just don't think that I can do it properly at home. At the doctor's office, she knows everything, so you know.” (Group 3).*

*“Yeah, because the doctor is professional and knows what they're doing. If it doesn't work, they can do it again on the spot. Obviously, we are not going to know more than them.” (Group 4).*

Participants were also concerned about the potential costs and time associated with being responsible for the self-collection, especially if the kit had to be purchased at a pharmacy or picked up at a clinic.

*“But if you put it on the shelf, some women may say ok I'll do it next week, or oh I don't have enough money right now, and they'll put it off. So honestly it goes back to it: give your bread to the baker. The doctor will just follow this [up] more.” (Group 4).*

*“I mean it defeats all purpose, why you are going to the clinic to begin with. You rather have the doctor do it and do it correctly.” (Group 2).*

*“But like having to go and buy it, sometimes it is kind of you know, it will like reduce that chances that people would take it.” (Group 2)*

### Increasing awareness

As previously noted, the participants cited a lack of awareness as a barrier to screening uptake. Participants stressed the need for tailored programming, especially for immigrant women. Several women mentioned the fact that they did not receive an adequate education about their health and well-being. The participants also noted the importance of discussing health issues with their children and modeling healthy behaviors from all members of the family.

Need for awareness: *“Women need awareness, education.” (Group 4).*

*“There is a lack of awareness in our Arab community, awareness is very weak. Although I say, thank God, we have progressed and have some light awareness but it's not very deep, there isn't anything about women's health and the health needs of women and their children. There just isn't an awareness that these things are important for women, maintaining health is important to let women live their lives, which could be taking care of her husband and raising her kids. But there is just no awareness, there needs to be more.” (Group 4).*

Modeling healthy behaviors: *“I can't tell my daughter to get the shot if I've neglected myself either. I have to be an example to them. When my daughter sees me taking care of myself, that is motivating. When I sit and talk to my son about how to use condoms, this is so he feels comfortable so that if something happens and things progress, he can come talk to me. Instead of going to his friend, or someone else who might give him bad advice. I don't know if you all agree with me, but this is a role that we have to play. From our house, then our neighbors, then our friends.” (Group 4).*

*“Really awareness is the most important thing, just like [Speaker 1] said. Especially as girls get older. Awareness is important and so is hygiene. It's nice for a girl – and even a boy – to get older and have understanding that things may harm them, like just understanding everything. You know, nobody informed us, but mothers have a role, fathers have a role, and even older siblings have one. Anyone who has information or knowledge has a role. (Group 3).*

### Fatalism

Among participants, there was a consensus that fatalism impacts the way their peers consider their health. The focus group participants posited that fatalism and a laissez-faire attitude toward health is an issue that prevents their peers from seeking care.

Fatalism: *“You can have a woman who is educated and aware, and she can meet with them and teach them about the disease and tell them they have to go get the Pap smears, and then a woman goes to her husband or her father and says, I need to have this done. They will reply saying "leave it to God." (Group 3).*

Laissez-faire attitude: *“Because they do not think it is going to happen to them, you know. I have my good friend: her mom was just diagnosed with breast cancer stage one. They have a history of cancer in their family and my friend who is in her thirties refuses to check. I told her, I am like, “You need to go check and make sure every year, especially because it runs in your family, and like every generation has had it.” So, she refuses to because she is like, “That is not going to happen to me. I am healthy.” So, I think that-, well I put it out there what my thoughts are. But that is my biggest concern with cancer in the community, just not thinking it is going to happen to them. And think most people think like that.” (Group 1).*

## Discussion

There is limited understanding of why screening rates for cervical cancer in the Arab American community are low. To address this gap in the literature, we carried out a series of focus groups with Arab American women residing in Michigan about their screening behaviors as well as the screening behaviors within their communities. Our study found that low cervical cancer screening rates in this population of Arab American women was due to multiple reasons: negative associations with screening, including discomfort and embarrassment; anxiety and difficulty making time to schedule appointments (termed by participants as neglect and fatalism); and a lack of cancer prevention awareness amongst the participants and community. Broadly, the women expressed mixed feelings about HPV self-collection such that it is not clear this approach, at least alone, will be successful in improving screening rates.

Our results help to flesh out cervical cancer screening views as there are sparse data on attitudes towards cervical cancer prevention amongst women from the Middle East and North Africa^11,24–30^. Two previous studies, one in Dearborn, Michigan, and one in New York City, focused on the attitudes of women who identify as Arab, Arab American, or Chaldean^25,31^. Similar to our findings, the KinKeeper study carried out in Dearborn, Michigan among women aged between 21 and 70^11,28–30^ identified lack of a doctor recommendation and competing interests as reasons for low screening uptake. Our participants indicated health literacy and awareness as barriers to cervical cancer screening on a community level which was not observed in the KinKeeper study among their Arab American participants. Further, unlike the KinKeeper study, fatalism was a common theme in our study and the study conducted in New York City among Arab American women.

Relatedly, three studies on cervical cancer screening have been carried out among Muslim women. The studies conducted in San Francisco and Chicago^24,27^ included a limited number of Arab and Arab Americans whereas the study in Pennsylvania exclusively explored the attitudes of Arab Muslims^26^. Similar to our findings, the San Francisco-based study which was carried out among those aged 18 and 25 identified family pressures and health care costs and access^24^. The study carried out in Pennsylvania also identified healthcare costs and access as barriers to screening uptake^26^. Conversely, the Chicago-based study carried out amongst Muslim Americans aged between 18 and 65 found that viewing health problems as a punishment from God was associated with lower uptake^27^, a theme that did not emerge in our study. Fatalism was not associated with cancer screening uptake in the Chicago study. The differences across studies could be due to the difference in populations: both our study and the New York study^25^ exclusively recruited Arab Americans, regardless of religion, whereas in Chicago, the participants were Muslims, regardless of ethnicity^27^.

Beyond barriers to screening, our study evaluated the acceptability of various HPV self-screening methods as an alternative to Pap tests, which has not yet been done in this population. The acceptability of alternative screening methods was mixed and likely would need to be scaffolded by health education efforts by trusted community organizations. Our study also evaluated attitudes towards HPV vaccination. Additionally, our study sheds light on a growing trend in the Arab American population: sentiments of vaccine hesitancy^32–34^. Vaccine hesitancy has strongly emerged as a topic warranting deep understanding in recent years, especially with the rise of the COVID-19 pandemic. This rising attitude has been documented amongst migrant groups globally and in the United States^35–41^. Because cervical cancer is a preventable disease through a combination of screening, treatment of precancerous lesions, and vaccination, efforts to understand vaccine hesitancy in Arab American communities is crucial. However, in the United States there is hesitancy overall towards the HPV vaccine^42^, as well as documented hesitation amongst immigrant groups^43–46^. To our knowledge, there has been no study evaluating vaccine hesitancy amongst Arab Americans, except for hesitancy to COVID-19 vaccination^33,34^. Further investigation is warranted to understand this phenomenon in the Arab American population.

Our study has several notable strengths. First, to our best knowledge, this is the first qualitative study to explore cervical cancer screening and HPV vaccination amongst women who identify as Arab, Arab American, and/or Chaldean women. Previous studies focused on Muslim women^24,27^ or Arab Muslim women^26^, which fails to account for the diversity of the Arab American population. Further, extrapolating results from Muslim women to describe reasons why Arab American women have poor screening is problematic as the majority of Muslim women in the United States are not Arab^47^.

While Michigan is an ideal place to study Arab American health due to the densely concentrated population in Southeastern Michigan^48^, Arab Americans live all over the United States and its territories^49^; as such, there may be significant differences between Arab Americans residing in California or New York as opposed to Michigan. For example, a general health-focused study carried out in New York amongst Arab American immigrants found that a lack of culturally competent care and concerns about discrimination and potential deportation were the biggest barriers to seeking cervical cancer screening^25^. However, these were not concerns shared by participants in our focus groups. This indicates that there may be geographically patterned reasons why Arab American women have poor uptake of cervical cancer screening.

The results of our study provide several exciting future directions for research. The attitudes towards vaccination, particularly vaccine hesitancy, warrant further exploration, especially as vaccination becomes a contentious subject in the United States. Though mixed, the overall positive attitudes towards HPV self-collection as a form of cervical cancer screening are also encouraging and indicate potential for piloting a self-collection method among Arab, Arab American, and/or Chaldean women to determine if self-collection could improve screening rates.

Lastly, our findings indicate a strong need for a focus on health awareness and health literacy amongst this population. Within the focus groups, various women expressed how they themselves tell their friends and family about the importance of cervical cancer screening and health maintenance. However, there was a consistent discussion about the need for a concerted effort to educate women of all ages within the Arab American community. Our findings also indicate that awareness campaigns and improvements in health literacy need to occur at the community level. Community-based interventions to improve health outcomes have been practiced with success amongst various minority populations. For example, a cluster randomized trial found that non-Hispanic Black men with uncontrolled hypertension had a reduction in blood-pressure when their barbers engaged in health promotion and encouraged their clients to participate in pharmacist-led interventions^50^. Additionally, a randomized trial found that community healthcare worker-facilitated HPV self-sampling led to increased cervical cancer screening amongst Haitian-American women^51^. Community workers, organizations, and centers, such as houses of worship and ethnic affinity centers, are uniquely positioned to engage in health awareness campaigns and interventions.

## Conclusions

Women who identify as Arab, Arab American, or Chaldean in Michigan identified many reasons why there is poor uptake of cervical cancer screening in their communities. The primary barriers they identified were a lack of health knowledge and health literacy. Through the focus groups, vaccine hesitancy also emerged as a potential barrier to cervical cancer prevention within this community.

## Data Availability

The participants of this study did not give written consent for their data to be shared publicly, so due to the sensitive nature of the research, supporting data is not available to be shared. If you have any questions or comments, please contact Lilah Khoja.
